# Tumor characteristics and survival rate of axillary metastatic breast cancer patients: a three decades retrospective cohort study

**DOI:** 10.1038/s41598-024-84115-7

**Published:** 2025-02-07

**Authors:** Seyed Mohamad Sadegh Mousavi-kiasary, Mahdis Bayat, Fereshteh Abbasvandi, Batoul Khoundabi, Fatemeh Mousavi, Atieh Akbari, Maryam Bagherian, Afsoon Zandi, Behnam Honarvar, Mohammad Esmaeil Akbari

**Affiliations:** 1 Health Policy Research Center, Institute of Health, Shiraz University of Medical Sciences, Shiraz, Iran; 2 Cancer Research Center, Shohadaye Tajrish Hospital, Shahid Beheshti University of Medical Sciences, Tehran, Iran; 3 ATMP Department, Breast Cancer Research Center, Motamed Cancer Institute, ACECR, Tehran, Iran; 4 Iran Helal Institute of Applied-Science and Technology, Red Crescent Society of Iran, Tehran, Iran; 5 Department of Hematology and oncology and stem cell transplantation, Firoozgar Hospital, School of Medicine, Iran University of Medical Sciences, Tehran, Iran; 6 Department of Otolaryngology, Head and Neck Surgery, Taleghani Hospital, Shahid Beheshti University of Medical Sciences, Tehran, Iran; 7 Department of Public Health, School of Public Health and Safety, Shahid Beheshti University of Medical Science, Tehran, Iran

**Keywords:** Breast cancer, Axillary lymph nodes, Disease-free survival, Overall survival rate, Breast cancer, Medical research

## Abstract

Background: Lymph node (LN) involvement, as an important prognostic factor in breast cancer (BC) patients, has a crucial role in their therapeutic approach. Consequently, a great desire is to thoroughly assess the patients based on their axillary LN status. The present study evaluated the characteristics and survival rate of axillary metastatic BC patients in a Tertiary and referral center. Method: The overall survival, disease-free survival, and clinicopathological characteristics of axillary metastatic BC patients referred to the Cancer Research Center in Tehran, Iran, from 1991 to 2022 were assessed retrospectively. We obtained patients’ clinical data from prospectively maintained registries. Result: Among the total 3399 recruited patients, 49.1%, 26.3%,13.1%, and 6.4% were pN0, pN1, pN2 and pN3, respectively. The pN0 group patients showed a significantly lower Hazard Ratio (HR) for DFS and OS compared to others. Moreover, estrogen and progesterone receptors, human epidermal growth factor2, tumor pathology type and tumor grade were prognostic factors of axillary LN status. Accordingly, pN0 patients had a lower recurrence risk than the others (*P* = 0.01). Conclusion: The axillary lymph node status has been considered as one of the fundamental factors determining the therapeutic strategy and prognosis of BC patients, which has an association with tumor characteristics. Regarding the crucial impact of the LN status on the survival landscape of breast cancer patients, accurate detection of the involved one and close screening follow-up of patients with more metastatic LNs during the surgery have a high value.

## Introduction

Breast cancer (BC) is the most common malignant tumor in women, which is two times more than other sites of solid tumors. Despite the advances made in the early detection and treatment of BC, the disease is one of the most common leading causes of cancer death in the worldwide^1^. BC has a broad spectrum of molecular, pathological and clinical features with diverse prognostic and therapeutic approaches, which axillary lymph node (LN) status is one of the critical independent prognostic predictors for disease-free survival (DFS) and overall survival (OS) of these patients^2–4^.

Regarding the significant impact of axillary LNs involvement in the therapeutic approach of BC patients, several studies focus on this field. Although, in past decades, clinicians believed that complete dissection of axillary LNs could lead to appropriate patient treatment, today’s research demonstrated that axillary lymph node dissection offers minimal or no oncological benefit for overall survival^5^. Therefore, the evolution of axillary surgery in the BC therapeutic approach has been driven mainly by a movement towards de-escalation of invasive interventions, thus leading to reduced surgery-associated morbidity and ensuring optimum patient quality of life while keeping oncological safety^6^. Recently, sentinel lymph node biopsy has become a standard method for staging most patients undergoing surgery to address the complete avoidance of axillary dissection surgery for certain BC patients^7^.

Regarding this opinion, predicting factors including clinical, radiologic, and pathologic features of breast tumors could be useful for selecting appropriate axillary surgery in BC patients^8^. Tumor characteristics such as immunohistochemistry (IHC), expression of steroid receptors, and human epidermal growth factor receptor 2 (HER2) status are highly specific for predicting axillary lymph node metastasis, though their prognostic impact has not been widely established^9^.

Considering this ongoing endeavor to identify axillary staging accurately, we aimed to determine the disease-free survival and overall survival of BC patients with axillary LN metastasis, also evaluating the correlation between the axillary LN status with immunohistochemistry features of breast tumors.

## Method

### Study design

In this study, the breast cancer patients diagnosed between April 1991 to March 2022 were identified from prospectively maintained breast cancer registries in the Cancer Research Center (CRC) in Tehran, Iran. Shahid Beheshti University of Medical Sciences and the respective ethics committees in the participating institution approved the study (ethics number: IR.SBMU.CRC.REC.1401.034), and all methods were performed in accordance with the relevant guidelines and regulations. The informed consent was obtained from all subjects and/or their legal guardian(s).

**Fig. 1:**
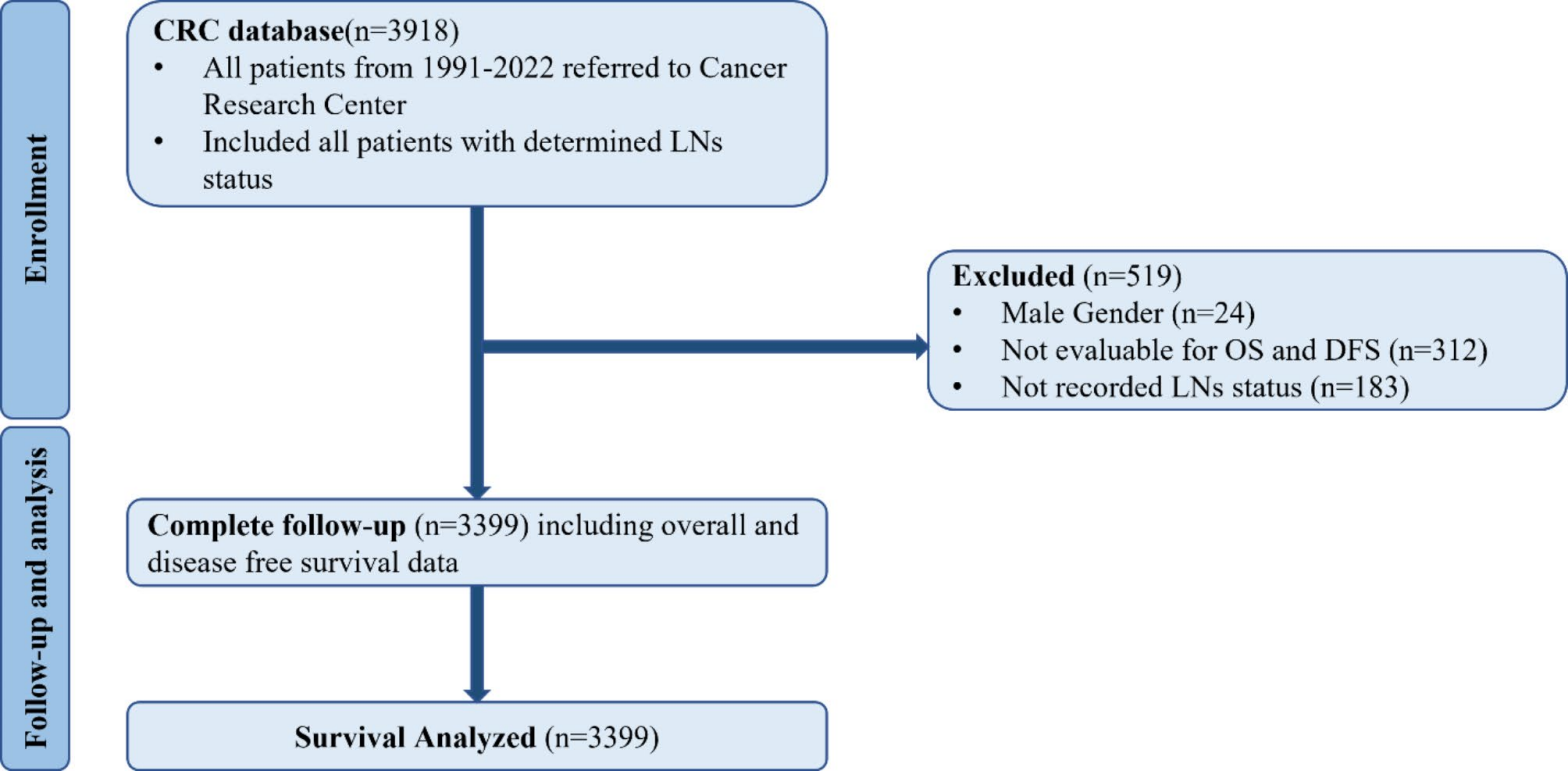
Consort of patients. CRC: Cancer Research Center. LNs: Lymph Nodes.

All the mentioned patients who underwent surgery were recruited in the study, and an experienced breast pathologist verified their diagnoses through histopathological examination. From a total of 3918 patients, the following patients were excluded (Fig. 1):

- Male patients.
- Patients with indeterminate or missing LN status.
- Patients with missing DFS and OS information (zero encounter/follow-up).

### Variables and outcome measure

In the current study, the demographic information (such as age, gender, and family history), tumor characteristics (including estrogen receptor (ER), progesterone receptor (PR), Ki67, tumor grade, stage, HER2 status), and treatment approach (chemotherapy, hormone therapy, and receiving trastuzumab) were obtained from the hospital information system. The TNM staging system of the BC patients was used to determine the tumor’s stage and LN status.

All the patients in this study underwent breast surgery, including mastectomy or breast-conserving surgery (BCS), also everyone received treatment in accordance with the latest standard guidelines for chemotherapy and radiotherapy. Physicians visit the patients at clinics and undergo regular follow-ups at the cancer research center or contact them via phone. In survival analysis, all the instances of cancer recurrence or mortality have been diligently documented, including the date and cause of events and cancer-related deaths. Cancer recurrence sites were also assessed up to 15 years of follow-up. In Table 1, the patient’s basic information is presented in detail.

### Statistical analysis

The data were analyzed using IBM SPSS ver. 27 (IBM Corp., Armonk, NY, USA) and Graph Pad version 9 (GraphPad Software, San Diego, CA, USA). The categorical variables are expressed as proportions and frequencies. The Kolmogorov-Smirnov test was applied to test the normality distribution. The quantitative variables with normal distribution are summarized as Mean ± Standard deviation (SD) and Median. To explore the impacts of categorical independent variables, the chi-square test was used. Mean values were compared between two groups using the independent or paired t-test, as well as using non-parametric tests, such as the Mann-Whitney U-test. In the analysis of variance, the Kruskal-Wallis test was applied to compare mean values among more than two groups. Kaplan–Meier analysis was used for univariate analysis, and log-rank tests were used for group comparisons. Cox proportional hazards regression was used for multivariate analysis (DFS and OS adjusted for age of diagnosis, family history, Herceptin use, ER status, PR status, grade, and recurrence state). Using scaled Schoenfeld residuals, the assumption of proportional hazard was checked for the final models. P-values less than 0.05 were considered to indicate statistical significance.

## Results

### Patients’ characteristics

There were 3918 patients with breast cancer in the database of the Cancer Research Center, among which 24 male patients, 183 patients with missing LNs status and 312 patients with missing DFS and OS information and no encounter after the first visit, were excluded. In total, 3399 patients have been included in the DFS and OS analysis. Table 1 provides the patients’ baseline characteristics for positive results across the number of lymph node statuses. Among included patients, 49.1%, 26.3%,13.1% and 6.4% of patients were with 0 (pN0 group), 1–3 (pN1 group), 4–9 (pN2 group) and greater and equals to 10 (pN3 group, it does not involve the presence of supraclavicular, infraclavicular or internal mammary lymph nodes.), positive lymph node number respectively. The differences in the patient mean age were insignificant between the groups (*P* = 0.075), and the pN0 group, with a mean age of 49.4, was the oldest. The frequency of positive ER and PR was more than negative among all the groups; however, the pN1 group had the highest proportion of ER and PR positive status. Regarding Ki67, the pN2 group had the highest Ki67 among the whole groups (28.41 ± 20.27), although the difference wasn’t significant between pairs of groups except it was substantial among the pN0 and pN2 (*P* = 0.034) and also it was significant among pN1 and pN2 (*P* = 0.040).

**Table 1:** Baseline patients’ characteristics.

Variable	Lymph node status				Overall	*P*-value
	0(*n* = 1758)	1–3(*n* = 942)	4–9(*n* = 471)	>=10(*n* = 228)		
Age, Mean ± SD Median	49.4 ± 11.69	48.31 ± 11.19	48.26 ± 11.44	48.52 ± 11.2	48.92 ± 11.54	0.075
	48	47	47	48	48	
Age Group						
<=50, n (%)	978(57.5)	562(61.8)	267(61.0)	121(57.9)	1928(59.2)	0.153
> 50, n (%)	722(42.5)	347(38.2)	171(39.0)	88(42.1)	1328(40.8)	
Family History, n (%)	1052(59.8)	576(61.1)	296(62.8)	139(61.0)	2063(60.7)	0.005
Ki67%, Mean ± SD Median	26.50 ± 23.06	25.11 ± 20.09	28.41 ± 20.27	26.08 ± 21.03	26.29 ± 21.81	0.150
	20	20	25	20	20	
ER status						
Positive, n (%)	1245(70.8)	726(77.1)	355(75.4)	147(64.5)	2473(72.8)	< 0.001
Negative, n (%)	507(28.8)	212(22.5)	113(24.0)	79(34.6)	911(26.8)	
Unknown, n (%)	6(0.3)	4(0.4)	3(0.6)	2(0.9)	15(0.4)	
PR Status						
Positive, n (%)	1142(65.0)	671(71.2)	318(67.5)	132(57.9)	2263(66.6)	< 0.001
Negative, n (%)	614(34.9)	266(28.2)	151(32.1)	94(41.2)	1125(33.1)	
Unknown, n (%)	2(0.1)	2(0.5)	2(0.4)	2(0.9)	11(0.3)	
Herceptin (No.), n (%)	1579(89.8)	818(86.8)	397(84.3)	194(85.1)	2988(87.9)	0.002
Hormone therapy (No.), n (%)	383(21.8)	155(16.5)	79(16.8)	52(22.8)	669(19.7)	0.012
Surgery						
BCS, n (%)	1363(77.5)	658(69.9)	272(57.7)	97(42.5)	2390(70.3)	< 0.001
Mastectomy, n (%)	352(20.0)	264(28.0)	191(40.6)	121(53.1)	928(27.3)	
Unknown, n (%)	43(2.4)	20(2.1)	8(1.7)	10(4.4)	81(2.4)	
Chemotherapy (No.), n (%)	322(18.4)	12(1.3)	2(0.4)	0(0.0)	336(9.9)	< 0.001
Recurrence/metastasis						
No recurrence/Metastasis, n (%)	1592(90.6)	808(85.8)	335(71.1)	146(64.0)	2881(84.8)	< 0.001
Locoregional Recurrence, n (%)	68(3.9)	41(4.4)	33(7.0)	10(4.4)	152(4.5)	
Bone metastasis, n (%)	27(1.5)	24(2.5)	26(5.5)	17(7.5)	94(2.8)	
Visceral and other sites metastasis, n (%)	71(4.0)	69(7.3)	77(16.3)	55(24.1)	272(8.0)	
Stage						
0, n (%)	5(0.3)	0(0.0)	0(0.0)	0(0.0)	5(0.0)	< 0.001
1, n (%)	659(37.5)	6(0.6)	0(0.0)	0(0.0)	665(19.6)	
2, n (%)	800(45.5)	696(73.9)	0(0.0)	0(0.0)	1496(44.0)	
3, n (%)	196(11.1)	226(24.0)	446(94.7)	217(95.2)	1085(31.9)	
4, n (%)	6(0.3)	13(1.4)	23(4.9)	11(4.8)	53(1.6)	
Unknown, n (%)	92(5.2)	1(0.1)	2(0.4)	0(0.0)	95(2.8)	
Grade						
1, n (%)	223(12.7)	69(7.3)	16(3.4)	9(3.9)	317(9.3)	< 0.001
2, n (%)	841(47.8)	510(54.1)	236(50.1)	93(40.8)	1680(49.4)	
3, n (%)	509(29.0)	308(32.7)	174(36.9)	103(45.2)	1094(32.2)	
Unknown, n (%)	185(10.5)	55(5.8)	45(9.6)	23(10.1)	308(9.1)	
HER2						
Negative, n (%)	1117(63.5)	553(58.7)	263(55.8)	112(49.1)	2045(60.2)	< 0.001
Low (1+/ 2 + not amplified), n (%)	235(13.4)	142(15.1)	66(14.0)	18(7.9)	461(13.6)	
Positive, n (%)	406(23.1)	247(26.2)	142(30.1)	98(43.0)	893(26.3)	
Pathology						
Invasive Ductal Carcinoma, n (%)	1512(86.0)	878(93.2)	433(91.9)	201(88.2)	3024(89.0)	< 0.001
Ductal Carcinoma In situ, n (%)	84(4.8)	0(0.0)	0(0.0)	0(0.0)	84(2.5)	
Invasive Lobular Carcinoma, n (%)	135(7.7)	44(4.7)	32(6.7)	23(10.2)	234(6.9)	
Other, n (%)	25(1.4)	19(2.0)	7(1.4)	4 (1.6)	54(1.6)	
ER/PR						
ER+/PR+, n (%)	1103(62.7)	654(69.4)	307(65.2)	123(53.9)	2187(64.3)	0.010
ER+/PR-, n (%)	142(8.1)	70(7.4)	48(10.2)	24(10.5)	284(8.4)	
ER-/PR+, n (%)	38(2.2)	17(1.8)	11(2.3)	9(3.9)	75(2.2)	
ER-/PR-, n (%)	469(26.7)	195(20.7)	102(21.7)	70(30.7)	836(24.6)	

**Fig. 2:**
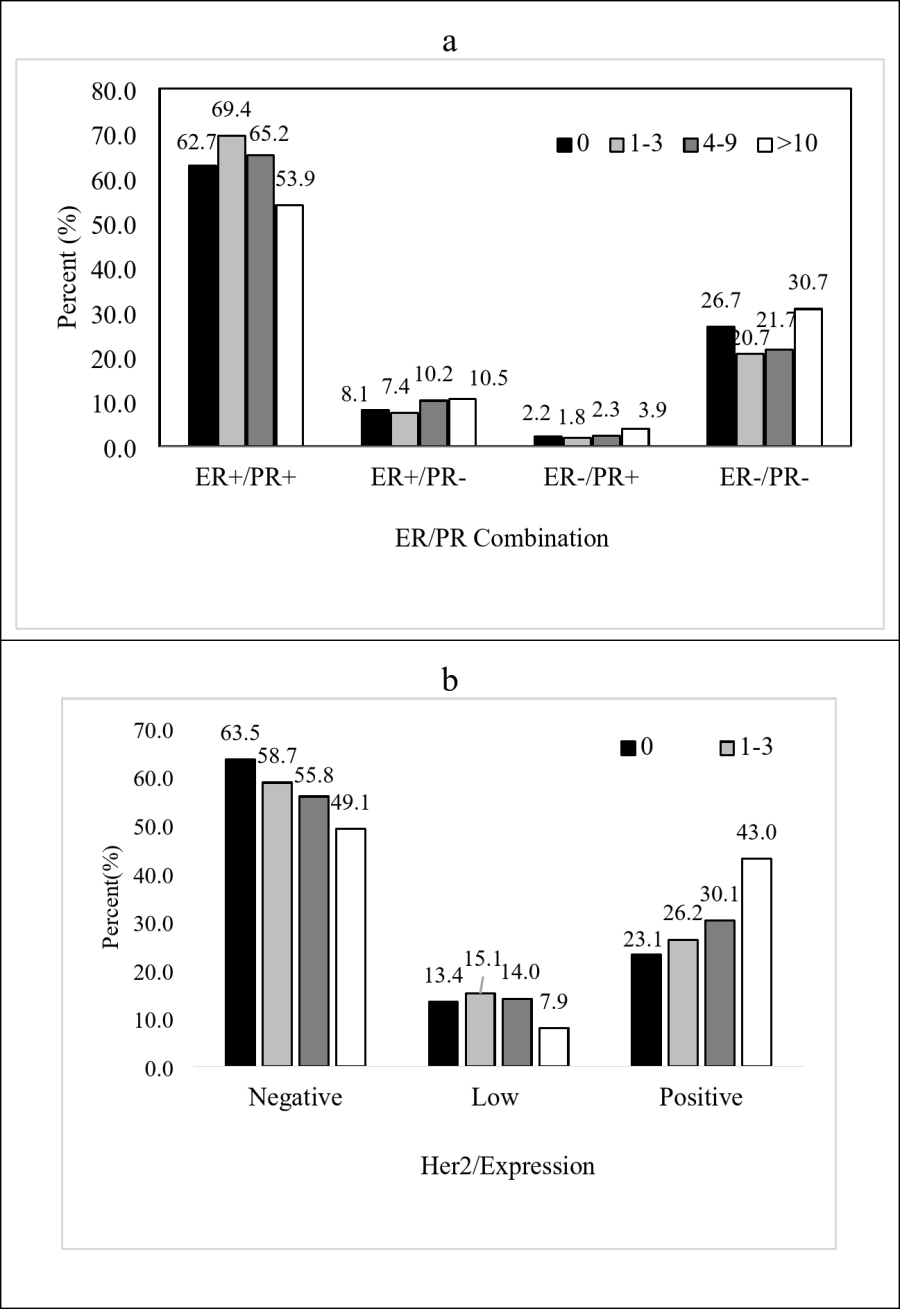
(**a**) Distribution of ER/PR expression and (**b**) HER2 expression with lymph node status in breast cancer patients. Distribution of cancer recurrence/metastasis site according to lymph node categories provided in Fig. 3. In the no recurrence/metastasis group, pN0 and pN2 groups, pN0 and pN3 groups, pN1 and pN2 groups and pN1 and pN3 groups were significantly different (*P* < 0.05). Furthermore, in the no recurrence/metastasis group, pN0 patients contain the majority of the patients, 90.6% (*P* = 0.01), while in visceral and other sites, metastasis pN3 patients are the most, 24.1% (*P* < 0.001).

The four possible combinations of ER and PR status: ER+/PR+, ER+/PR-, ER-/PR + and ER-/PR- were represented in four groups of lymph node numbers in Fig. 2a. There is a significant difference between the pN3 group with pN1 and pN0 groups, 53.9% versus 69.4 and 62.7%, respectively, in ER+/PR + proportion (*P* < 0.05). The proportion of ER-/PR- in pN3 and pN1 group patients has the highest and lowest, 30.7 and 20.7, respectively (*P* < 0.05). Figure 2b shows the distribution of lymph nodes by HER2 categories. HER2-negative was defined as the IHC score of 0, HER2-positive was defined as the IHC score of 3 + or IHC score of 2 + and in situ hybridization (ISH)-positive, while patients with IHC score of 1 + or 2+/ISH-negative were defined as HER2-low. HER2-negative proportion is significantly different between pN0 with pN2 and pN3 group, 63.5% versus 55.8% and 49.1% (*P* < 0.05). HER2-low proportion is also different between pN3 and the other three groups, 7.9% versus 13.4%, 15.1% and 14.0%, respectively (*P* < 0.05). HER2-positive proportion in the pN0 group significantly differs between pN0 and pN2 and pN3 with 23.1% versus 30.1% and 43.0%. The pN1 and pN2 groups are also different as HER2-positive proportion with pN3, 26.2% and 30.1% versus 43.0%.

**Fig. 3:**
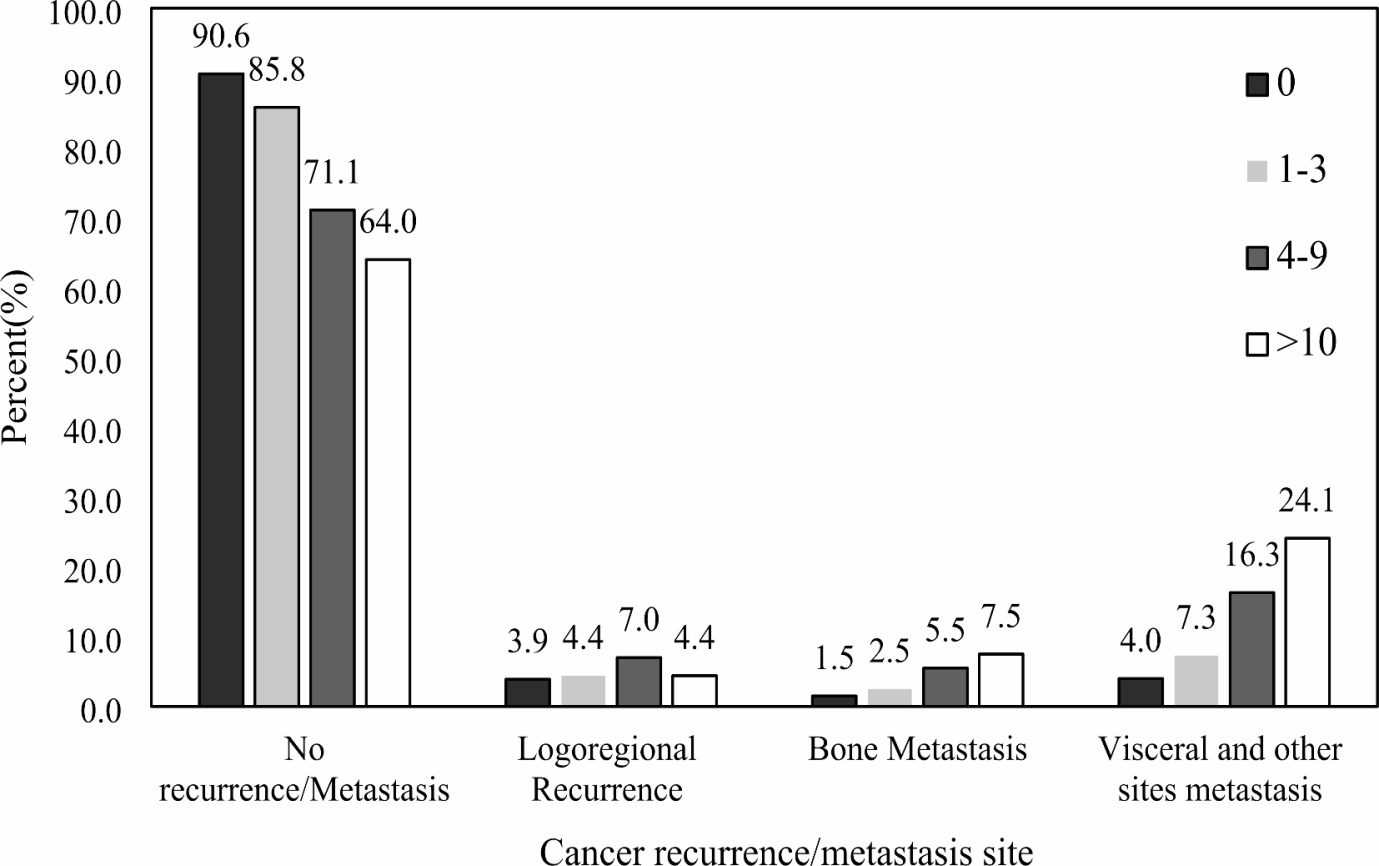
Cancer recurrence/metastasis site according to positive lymph node categories.

### Free-disease survival outcomes

**Fig. 4:**
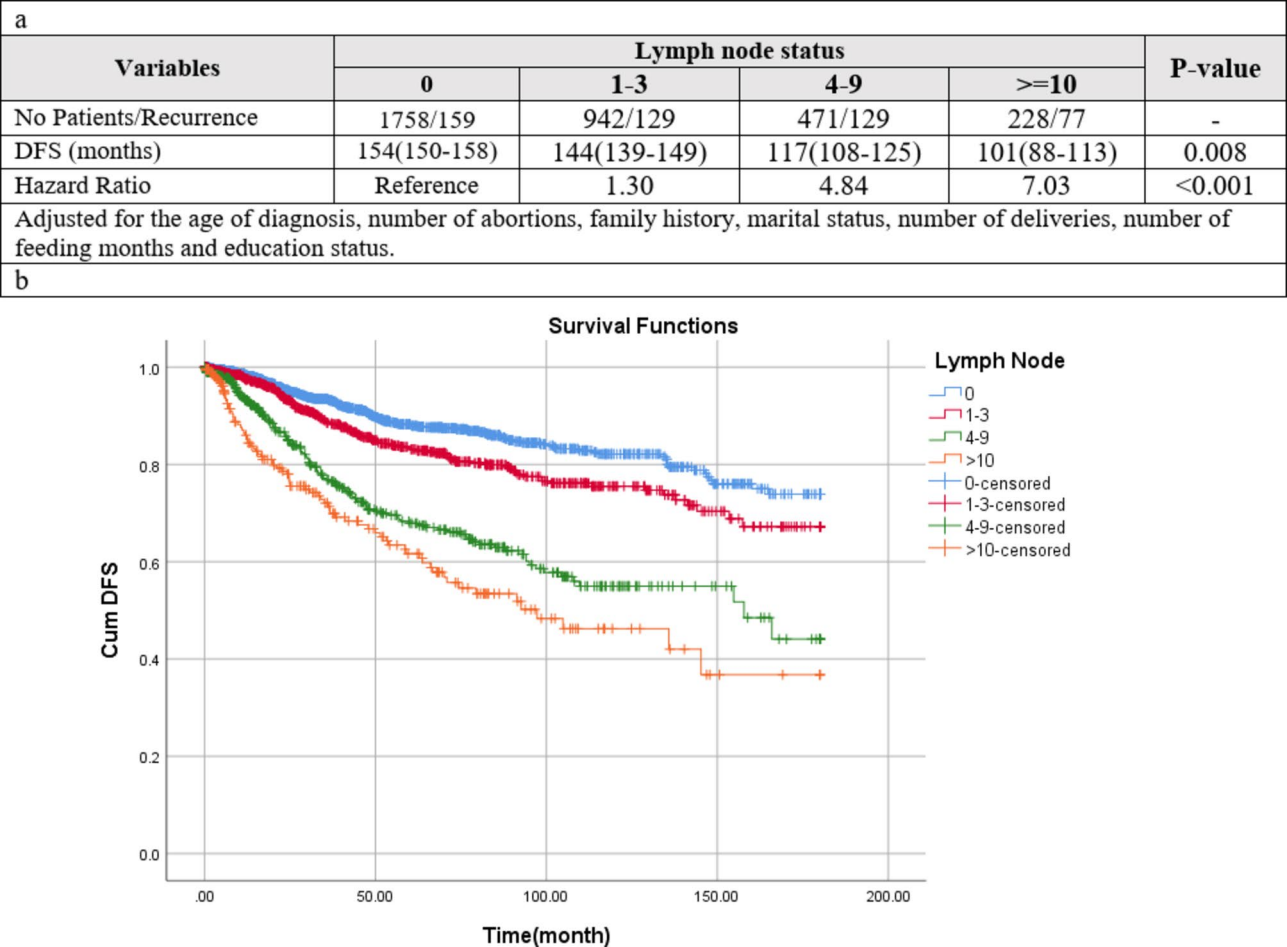
(**a**) Disease-free survival for different lymph node status. (**b**) Kaplan–Meier curves of disease-free survival (DFS) among patients according to lymph node status (P-value < 0.001)

The median follow-up of 3399 patients included in the DFS analysis was 33 (IQR 10–76) (mean 48.8; 95% CI 47.3–50.4) months. Altogether, 514 (14.3%) DFS events were observed among patients. Based on Kaplan–Meier analysis, the pN0 group had the highest mean of DFS (154 months), while the pN3 had the lowest duration of DFS (101 months) (Fig. 4a). Based on Cox model regression adjusted for the age of diagnosis, number of abortions, family history, marital status, number of deliveries, number of feeding months and education status, compared to pN0 group patients, other groups had a significantly higher hazard for recurrence in DFS. Compared to pN0 group patients, pN1, pN2 and pN3 patients had a significantly higher Hazar Ratio (HR) for DFS (HR 1.30, 95% CI 1.14–2.64), (HR 4.84, 95% CI 2.86–6.01) and (HR 7.03, 95% CI 3.29–9.94). For example, the patients with 4–9 positive lymph node numbers have 4.84 times the hazard of recurrence compared to the patients without positive lymph. In all lymph node statuses, the 5-year, 10-year, and 15-year DFS rates were 80%, 73%, and 64%, respectively. In detail, the mentioned DFS rates in the pN0 group were 86%, 81%, and 77%, respectively; in the pN1 group, they were 83%, 77%, and 70%; in the pN2 group, they were 70%, 68% and 58% and in final they were 63%, 44%, and 39%, in the pN3 patients (Fig. 4b).

### Overall survival outcomes

**Fig. 5:**
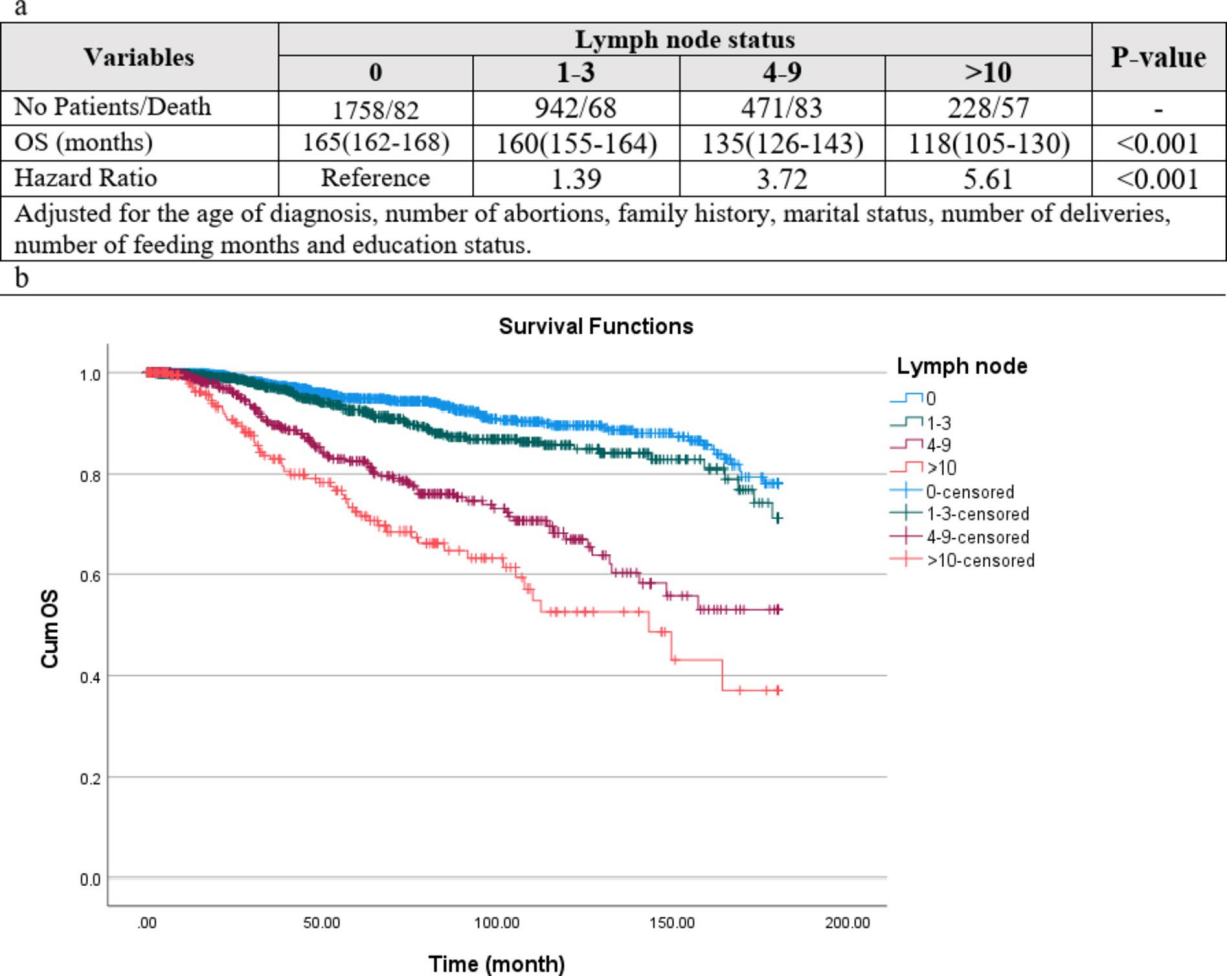
(**a**) Overall survival of the patients based on Lymph node. HR for pN1, pN2 and pN3 patients rather than pN0 group is respectively: (HR 1.39, 95% CI 1.03–2.01), (HR 3.72, 95% CI 2.77–5.33), (HR 5.61, 95% CI 3.99–8.07). For example, the patients with 4-9 positive lymph node numbers have 3.72 times the hazard of death compared to the patients without positive lymph. (**b**) Kaplan–Meier overall survival curves (OS among patients according to Lymph node status (P-value of Log-rank test < 0.001).

The median follow-up of 3399 patients included in the OS analysis was 38 (IQR 12–81) (mean 52.5; 95% CI 50.9–54.1) months. During follow-up, 310 (8.7%) deaths occurred among patients. The pN0 group had the highest mean of OS at 165 months, and the pN3 had the lowest duration of OS at 118 months, and a P value less than 0.001 shows the significance of this difference (Fig. 5a). Based on Kaplan–Meier analysis, the pN0 group had the highest mean of OS at 165 months, and the pN3 had the lowest duration of OS at 118 months, and a P value less than 0.001 shows the significance of this difference (Fig. 5b). Based on Cox model regression adjusted for the age of diagnosis, number of abortions, family history, marital status, number of deliveries, number of feeding months and education status, compared to pN0 group patients, all three groups, pN1, pN2 and pN3, had significantly higher hazard for death in OS. All patients’ 5-year, 10-year, and 15-year OS rates were 89%, 80%, and 69%, respectively. To be more precise, in the pN0 group, it was 96%, 89%, and 82%, respectively; in the pN1 group it was 93%, 83%, and 80%, respectively, and it was 81%, 72%, and 57%, in the pN2 patients and for pN3 was 70%, 55% and 38% (Fig. 5b).

### Multivariate analysis

Table 2 contains the results of multivariate Cox regression analysis adjusted for the age of diagnosis, ER and PR, grade, stage, hormone therapy, chemotherapy, recurrence, number of abortions, family history, marital status, number of deliveries, number of feeding months and education status for OS and DFS. As seen, grade, marital status and education have significant effect on DFS. Patients with grade 3, are 1.73 times more at risk of recurrence than those with grade 2 (*P* = 0.036). Married patients’ recurrence hazard is 0.55 times that of single patients (*P* = 0.039). The recurrence risk of patients with primary and secondary education is 1.79 times that of patients with university education (*P* = 0.019). The hazard of recurrence of illiterate patients is also 1.90 times that of patients with university education (*P* = 0.046).

On the other hand, OS is significantly influenced by some factors such as age of diagnosis, family history, stage, recurrence status, marital and education status. Patients aged 50 and over are 1.65 times more at risk of death than patients under 50 years of age (*P* = 0.029). Family history also significantly increases the risk of death by 1.34 times (*P* = 0.041). The death rate of patients with stage 3 or 4, is 3.37 times higher than that of patients with lower stages (*P* = 0.037). Recurrence leads to a death hazard 10.34 times rather than patients without recurrence experience (*P* < 0.001).

The hazard of death of married patients is 0.57 times that of single patients (*P* = 0.039). The hazard of death of patients with primary and secondary education is 1.86 times that of patients with university education (*P* = 0.029). The hazard of death of illiterate patients is 1.97 times that of patients with university education (*P* = 0.033).

**Table 2:** Multivariate analysis of patients’ characteristics, hazard ratio of their disease-free survival, and overall survival.

Variables	Multivariate analysis HR (95% HR)	
	Diseases free survival	Overall survival
Age		
< 50	1	1
>=50	0.89(0.74–1.07)	1.65(1.31–2.08) *
**PR**		
Negative	1	1
Positive	0.79(0.59–1.06)	0.70(0.47–1.05)
**ER**		
Negative	1	1
Positive	0.81(0.58–1.14)	1.04(0.70–1.53)
**Family History**		
No	1	1
Yes	0.91(0.75–1.10)	1.34(1.06–1.69) *
**Number of Abortion**		
0	1	
1	1.051(0.631–1.287)	1.033(0.599–1.308)
**Grade**		
1	1	1
2	1.46(0.95–2.26)	0.98(0.56–1.71)
3	1.73(1.11–2.69) *	1.33(0.75–2.34)
**Stage**		
0–1	1	1
2	1.21(0.87–1.68)	1.60(0.97–2.64)
3–4	3.60(2.63–4.94)	3.37(2.08–5.47) *
**Hormone Therapy**		
No	1	1
Yes	1.01(0.74–1.39)	0.82(0.55–1.22)
**Chemotherapy**		
No	1	1
Yes	1.56(1.19–2.61)	1.69(0.57–3.54)
**Recurrence**		
No	-	1
Yes	-	10.34(7.91–13.53) *
**Marital Status**		
Single	1	
Married	0.55(0.34–0.97) *	0.57(0.33–0.99) *
Divorced	0.302(0.066–1.108)	0.26(0.08–1.23)
Widowed	0.789(0.324–1.729)	0.93(0.57–2.53)
**Education**		
University	1	
Diploma	1.089(0.708–1.429)	1.12(0.80–1.51)
Primary & Secondary	1.79(1.25–2.89) *	1.86(1.13–2.64) *
Illiterate	1.90(1.07–3.46) *	1.97(1.09–3.68) *
**Number of Delivery**	1.11(0.98–1.24)	1.09(0.98–1.23)
**Number of Feeding (Month)**	0.99(0.98-1.00)	1.01(0.99–1.09)

### Patients without positive lymph node

**Fig. 6:**
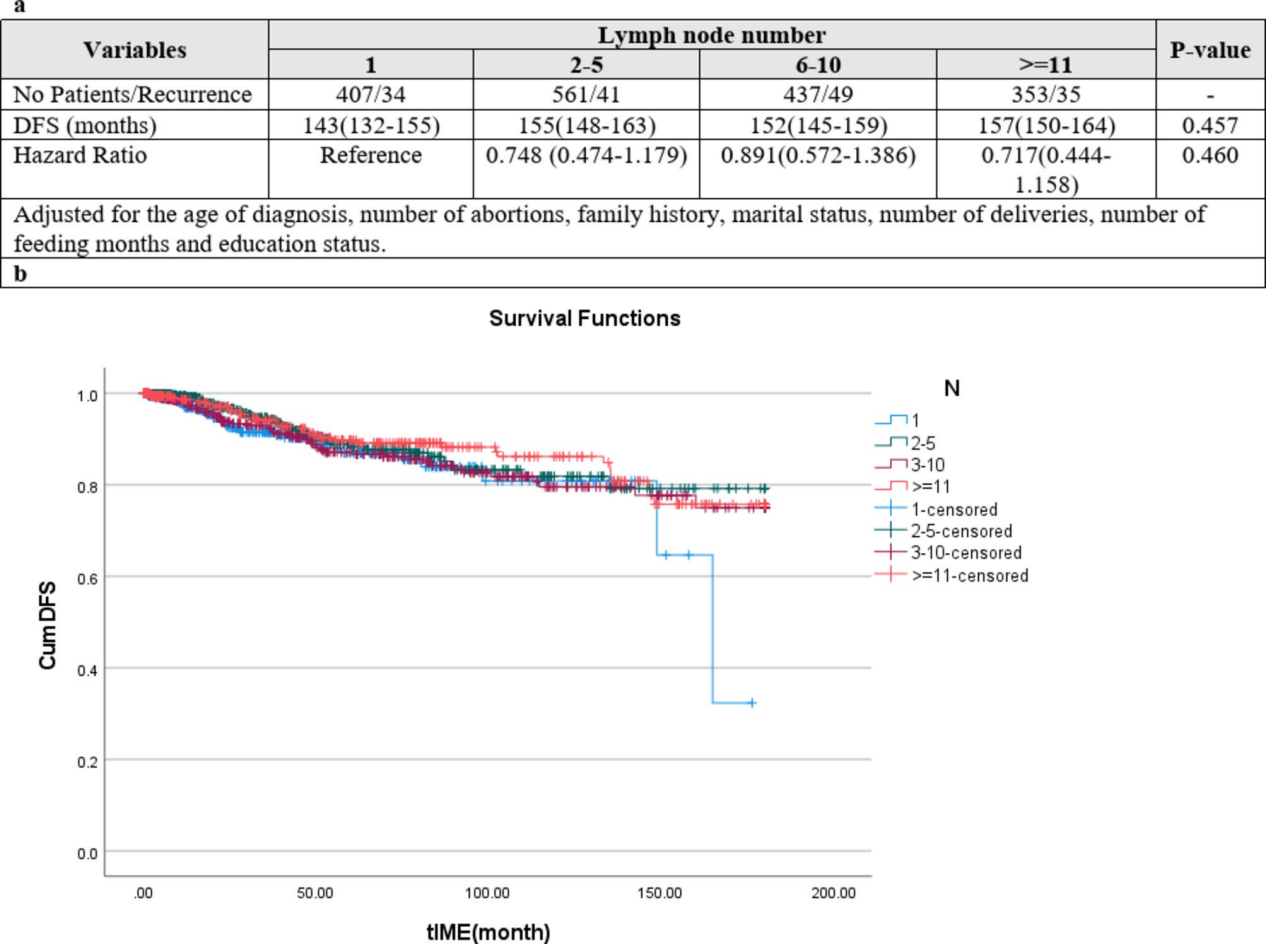
(**a**) Disease-free survival for different lymph node numbers. (**b**) Kaplan–Meier curves of disease-free survival (DFS) among patients according to lymph node number (P-value < 0.001)

**Fig. 7:**
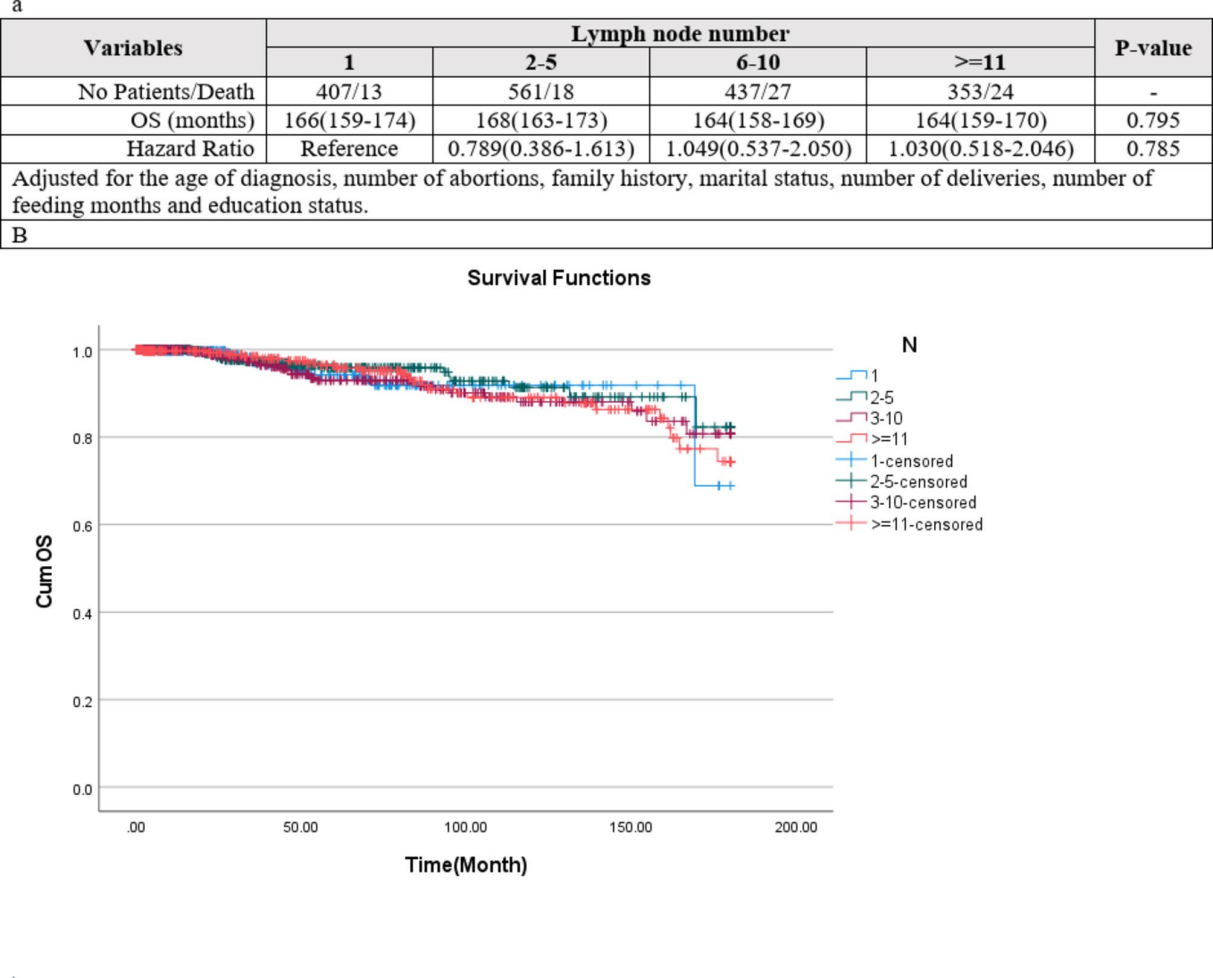
(**a**) Overall survival of the patients based on Lymph node number. (**b**) Kaplan–Meier overall survival curves (OS among patients according to Lymph node status (P-value of Log-rank test < 0.001).

The hazard of recurrence for DFS and hazard death for OS in different categories of lymph node numbers containing 1, 2–5, 6–10 and more than 11 was assessed and compared in the following. Figures 6 and 7 show the results of Kaplan–Meier and multivariate Cox regression analysis adjusted for the age of diagnosis, number of abortions, family history, marital status, number of deliveries, number of feeding months and education status, respectively, for DFS (Fig. 6 ) and OS (Fig. 7 ). The mean of DFS and hazard of recurrence had no significant difference at 0.05 level (Fig. 6a and b). The mean of OS and hazard of death had no significant difference at 0.05 level (Fig. 7a and b).

## Discussion

In the present large-scale cohort study, we have evaluated the epidemiological and survival characteristics of axillary metastatic breast cancer patients, along with the free axillary LN group, in a total of 3399 patients. Taken altogether, disease-free and overall survival rates were significantly better among pN0 patients compared to other groups. This finding would prove the idea that axillary LN involvement could be a valuable prognostic factor in breast cancer patients. The total proportion of the pN0 group among the 3399 patients in our study was 49.1%; in addition, about 26.3%, 13.1% and 6.4% of the patients were pN1, pN2 and pN3, respectively. Taken together, 50.9% of the patients in this cohort had at least one axillary LN metastasis. This observed result could be an indicator of the high incidence of axillary LN metastasis in our country. In our cohort, the mean age of all participants was 48.92 years (SD, 11.54), which was near the results of two other studies in 2008 and 2014^9,10^. However, this was lower than the reported result in another study in 2009 (62.7 years (SD, 13.8))^11^. This observed difference could be due to various sampling or sample sizes, as well as possible variations in the genetic background of patients in distinct countries.

To investigate the risk factors of axillary LN metastasis of BC patients, a variety of nomograms such as MSKCC Nomogram^12^, Cambridge Nomogram^13^, and Mayo Nomogram^14^ have been designed which have high reference value for clinical application. These nomogram models contain various predictive factors like MSKCC columns, which consider age, tumor location, tumor size, multifocality, tumor type, vascular invasion, histological grade, and ER and PR status for axillary LN involvement. A series of studies reported that these biomarkers could provide reliable predictive analysis for evaluating axillary LN metastasis^15^. The present study suggested that tumor pathology, tumor grade, and ER, PR and HER2 status can effectively predict axillary LN involvement in patients with invasive breast cancer. In terms of tumor pathology, in the present study, the most frequent histological subtype was invasive ductal carcinoma (IDC) (89%), the same as Ali’s study^9^, and the rate of involved LNs reported higher in the IDC group compared to invasive lobular carcinoma (ILC) one which could be owing to the low proportion of ILC tumors in our recruited patients. Regarding the grade of the tumor, in pN0 patients, about 12.7% were in grade I, while only 3.9% were low grade in pN3 patients. In line with this observation, multiple past studies demonstrated that higher tumor grades are significantly associated with axillary LN metastasis^9,16–18^.

Taking the hormone receptor status of breast tumors into consideration, our results showed a significantly higher frequency of hormone receptor (ER and PR) positivity in the pN0 group compared to the others, in agreement with several previous studies^9,19–21^. In contrast, a couple of other studies reported no value for ER and PR as a valid predictor for axillary lymph node status^22–24^. Since HER2 status has been found to be one of the adverse prognostic factors of BC patients, HER2 positive tumors are characterized by vigorous cell proliferation, leading to the invasion and metastasis of BC^25^. Consistent with the previous studies, the obtained results showed a significant correlation between HER2 overexpression and axillary LN involvement^9^. On the other side, in other research studies, there was no significant association between HER2 status with presence of axillary LN metastasis^24,26,27^. Taken altogether, it seems that clinicopathological characteristics of the breast tumor need to be considered as a predisposing factor of axillary lymph node status.

In the current study, pN3 patients showed the lowest DFS and OS among the whole patients. The differences were significant between pN0 and the other three groups. This is consistent with another study in 2008, in which they found that the percent of positive nodal involvement could be a significant prognostic factor for survival rates of breast cancer patients^28^. In line with the aforementioned data, another study in 2011 elucidated that breast cancer patients with metastasis of ten or more lymph nodes have poor prognosis in comparison to patients with less LN involvement^29^. All in all, regarding the survival rate of BC patients based on the axillary LN status, our data showed significantly better DFS and OS rates of pN0 compared to other groups which involved axillary LNs.

As previously mentioned, axillary lymph node status has a highly significant impact on a breast cancer patient’s prognosis. In the present study, we found out that the pN3 group had a significantly higher rate of visceral and other (than locoregional and bone metastasis) recurrence sites; consistently, in no recurrence category, the pN0 group was considerably higher than the pN2 and pN3 participants. Related to recurrence, Costeira et al. reported that metastatic axillary lymph nodes are one of the important factors associated with breast cancer locoregional recurrence^30^.

Moreover, it’s noteworthy to depict that assessment of the demographic information of the patients indicated that family history, education and marriage status, along with other clinical factors including pathology of tumor and grade, had a significant impact on the survival rate of the patients. For more elaboration, married patients and cases with higher education degree have shown better DFS and OS. Although, pregnancy history, abortion, and breastfeeding showed no statistical difference in the survival rate of the BC patients. In consistent in 2020, the study reported that Age, education and financial situation, and marital status had an impact on the quality of life of breast cancer patients^31^. Also, in another study in 2012 found out that late presentation of the disease was associated with marital status, level of education, lack of benign breast illness history and menopausal age^32^. Regarding these results, besides the clinicopathological presentation, the demographic characteristics of the patients could enhance their prognosis of them and justify the necessity to promote public awareness. In addition, considering these findings may lead to increasing efforts to establish comprehensive breast cancer control programs in Iran.

The limitations of this study, including missing patient information, lack of precise records of LN status, reliance on a single-center approach, absence of genetic background data, and not considering the other predictors for axillary LN status as tumor location in the breast, tumor vascularization and lymph angiogenesis, collectively have the potential to impact the study’s outcomes. Moreover, as chemotherapy treatments have changed over the years, the patients are not comparable in terms of response or prognosis. To address these limitations in future research, including through prospective multicenter studies with genetic information, which can improve the application of the study.

## Conclusion

In conclusion, this study showed a significantly better survival rate and less aggressive traits in patients without axillary LN metastasis than in participants with involved ones. Our data lends credence to the idea that tumor characteristic features are high significant representation of axillary LN status. Furthermore, the demographic information of the patients including their family history, education and marriage status, had an impact on the patients’ survival rate. Although, pregnancy history, abortion, and breastfeeding showed no statistical difference in OS and DFS of the BC patients. Considering these features may enhance the detection rate of involved LNs, thus reducing unnecessary axillary lymph node dissection. The axillary LN status has a crucial impact on the survival landscape of breast cancer patients, and accurate detection of the involved one will also present a tailored approach for patients who underwent surgery. Regarding this, close screening follow-up of patients with more metastatic LNs during the surgery is highly valued.

## Data Availability

The datasets used and analyzed during the current study are available from the corresponding author upon reasonable request .

## References

[1] Li, N. et al. Global burden of breast cancer and attributable risk factors in 195 countries and territories, from 1990 to 2017: Results from the global burden of disease study 2017. *J. Hematol. Oncol.* **12** (1), 1–12 (2019). 31864424 10.1186/s13045-019-0828-0 PMC6925497

[2] Dubsky, P. et al. Breast conservation and axillary management after primary systemic therapy in patients with early-stage breast cancer: The Lucerne toolbox. *Lancet Oncol.* **22** (1), e18–e28 (2021). 33387500 10.1016/S1470-2045(20)30580-5

[3] Hotton, J. et al. Pre-operative axillary ultrasound with fine-needle aspiration cytology performance and predictive factors of false negatives in axillary lymph node involvement in early breast cancer. *Breast Cancer Res. Treat.* **183**, 639–647 (2020). 32737710 10.1007/s10549-020-05830-z

[4] Fujii, T. et al. Implication of 18F-fluorodeoxyglucose uptake of affected axillary lymph nodes in cases with breast cancer. *Anticancer Res.* **36** (1), 393–397 (2016). 26722071

[5] Gatzemeier, W. & Mann, G. B. Which sentinel lymph-node (SLN) positive Breast cancer patient needs an axillary lymph-node dissection (ALND)–ACOSOG Z0011 results and beyond. *Breast* **22** (3), 211–216 (2013). 23478200 10.1016/j.breast.2013.02.001

[6] Thomssen, C. et al. International consensus conference for advanced breast cancer, Lisbon 2019: ABC5 consensus–assessment by a German group of experts. *Breast Care*. **15** (1), 82–95 (2020). 32231503 10.1159/000505957 PMC7098316

[7] Žatecký, J. et al. Level I axillary dissection in patients with breast cancer and tumor-involved sentinel lymph node after NAC is not sufficient for adequate nodal staging. *Turkish J. Surg.* **39** (1), 1 (2023). 10.47717/turkjsurg.2023.5984 PMC10234716 37275927

[8] Wasuthit, Y. et al. Predictive factors of axillary lymph node metastasis in breast cancer. *J. Med. Assoc. Thai.* **94** (1), 65 (2011). 21425730

[9] Ali, E. M., Ahmed, A. R. & Ali, A. M. Correlation of breast cancer subtypes based on ER, PR and HER2 expression with axillary lymph node status. *Cancer Oncol. Res.* **2** (4), 51–57 (2014).

[10] Ayadi, L. et al. Correlation of HER-2 over-expression with clinico-pathological parameters in Tunisian breast carcinoma. *World J. Surg. Oncol.* **6**, 1–8 (2008). 18945339 10.1186/1477-7819-6-112 PMC2577672

[11] Onitilo, A. A. et al. Breast cancer subtypes based on ER/PR and Her2 expression: Comparison of clinicopathologic features and survival. *Clin. Med. Res.* **7** (1–2), 4–13 (2009). 19574486 10.3121/cmr.2009.825 PMC2705275

[12] Bevilacqua, J. L. B. et al. Doctor, what are my chances of having a positive sentinel node? A validated nomogram for risk estimation. *J. Clin. Oncol.* **25** (24), 3670–3679 (2007). 17664461 10.1200/JCO.2006.08.8013

[13] Pal, A. et al. A model for predicting non-sentinel lymph node metastatic disease when the sentinel lymph node is positive. *J. Br. Surg.* **95** (3), 302–309 (2008). 10.1002/bjs.5943 17876750

[14] Degnim, A. C. et al. Nonsentinel node metastasis in breast cancer patients: Assessment of an existing and a new predictive nomogram. *Am. J. Surg.* **190** (4), 543–550 (2005). 16164917 10.1016/j.amjsurg.2005.06.008

[15] Scow, J. S. et al. Assessment of the performance of the Stanford Online calculator for the prediction of nonsentinel lymph node metastasis in sentinel lymph node-positive breast cancer patients. *Cancer Interdiscip. Int. J. Am. Cancer Soc.* **115** (18), 4064–4070 (2009). 10.1002/cncr.24469 19517477

[16] Patani, N., Dwek, M. & Douek, M. Predictors of axillary lymph node metastasis in breast cancer: A systematic review. *Eur. J. Surg. Oncol. (EJSO)*. **33** (4), 409–419 (2007). 17125963 10.1016/j.ejso.2006.09.003

[17] Xie, F. et al. A logistic regression model for predicting axillary lymph node metastases in early breast carcinoma patients. *Sensors* **12** (7), 9936–9950 (2012). 23012578 10.3390/s120709936 PMC3444135

[18] Yoshihara, E. et al. Predictors of axillary lymph node metastases in early breast cancer and their applicability in clinical practice. *Breast* **22** (3), 357–361 (2013). 23022046 10.1016/j.breast.2012.09.003

[19] Nouh, M. A. et al. Lymph node metastasis in breast carcinoma: Clinicopathological correlations in 3747 patients. *J. Egypt. Natl. Canc Inst.* **16** (1), 50–56 (2004). 15716998

[20] Li, L. & Chen, L. Z. Factors influencing axillary lymph node metastasis in invasive breast cancer. *Asian Pac. J. Cancer Prev.* **13** (1), 251–254 (2012). 22502679 10.7314/apjcp.2012.13.1.251

[21] Bauer, K., Parise, C. & Caggiano, V. Use of ER/PR/HER2 subtypes in conjunction with the 2007 St Gallen Consensus Statement for early breast cancer. *BMC Cancer*. **10**, 1–12 (2010). 20492696 10.1186/1471-2407-10-228 PMC2886044

[22] Gajdos, C., Tartter, P. I. & Bleiweiss, I. J. Lymphatic invasion, tumor size, and age are independent predictors of axillary lymph node metastases in women with T1 breast cancers. *Ann. Surg.* **230** (5), 692 (1999). 10561094 10.1097/00000658-199911000-00012 PMC1420924

[23] Chua, B. et al. Frequency and predictors of axillary lymph node metastases in invasive breast cancer. *ANZ J. Surg.* **71** (12), 723–728 (2001). 11906387 10.1046/j.1445-1433.2001.02266.x

[24] Chen, H. et al. Correlation analysis of pathological features and axillary lymph node metastasis in patients with invasive breast cancer. *J. Immunol. Res*. **2022** (2022). 10.1155/2022/7150304 PMC9553448 36249424

[25] Kustić, D. et al. Impact of HER2 receptor status on axillary nodal burden in patients with non-luminal A invasive ductal breast carcinoma. *Rev. Med. Chil.* **147** (5), 557–567 (2019). 31859887 10.4067/S0034-98872019000500557

[26] Prati, R. et al. Histopathologic characteristics predicting HER-2/neu amplification in breast cancer. *Breast J.* **11** (6), 433–439 (2005). 16297088 10.1111/j.1075-122X.2005.00125.x

[27] Almasri, N. M. & Hamad, M. A. Immunohistochemical evaluation of human epidermal growth factor receptor 2 and estrogen and progesterone receptors in breast carcinoma in Jordan. *Breast Cancer Res.* **7**, 1–7 (2005). 16168103 10.1186/bcr1200 PMC1242123

[28] Lale Atahan, I. et al. Percent positive axillary lymph node metastasis predicts survival in patients with non-metastatic breast cancer. *Acta Oncol.* **47** (2), 232–238 (2008). 17924207 10.1080/02841860701678761

[29] Lee, J. S. et al. Factors influencing the outcome of breast cancer patients with 10 or more metastasized axillary lymph nodes. *Int. J. Clin. Oncol.* **16**, 473–481 (2011). 21360123 10.1007/s10147-011-0207-5

[30] Costeira, B. et al. Long-term locoregional recurrence in patients treated for breast cancer. *Breast Cancer Res. Treat.* **202** (3), 551–561 (2023). 37707638 10.1007/s10549-023-07089-6

[31] Konieczny, M. et al. Quality of life of women with breast cancer and socio-demographic factors. *Asian Pac. J. Cancer Prevent. APJCP*. **21** (1), 185 (2020). 10.31557/APJCP.2020.21.1.185 PMC7294011 31983183

[32] Ibrahim, N. & Oludara, M. Socio-demographic factors and reasons associated with delay in breast cancer presentation: a study in Nigerian women. *Breast* **21** (3), 416–418 (2012). 22381153 10.1016/j.breast.2012.02.006

